# Water and Gas Exchange Responses of Five Related Blueberry Species (Ericaceae spp.) to the Dry Season in the High‐Andean Forest of the Colombian Eastern Cordillera

**DOI:** 10.1002/pei3.70177

**Published:** 2026-07-06

**Authors:** Carolina Ramos‐Montaño, N. Milena Cárdenas‐Avella, Ledis V. Montenegro‐Rubiano, Angélica del Pilar Dallos, Rafael Andrés Rodríguez‐Sabogal

**Affiliations:** ^1^ Grupo Ecología de Organismos, Facultad de Ciencias Básicas, Centro de Laboratorios LS‐406 Universidad Pedagógica y Tecnológica de Colombia Tunja Boyacá Colombia; ^2^ Laboratório de Ciências Ambientais Universidade Estadual Do Norte Fluminense Darcy Ribeiro Campos dos Goytacazes RJ Brazil

**Keywords:** Andean blueberries, climate change, physiology, rainfall, soil water tension, stem water potential, water use efficiency

## Abstract

*Vaccinium*, *Cavendishia*, and *Macleania* are typical genera of blueberries from the Ericaceae belt, a narrow ecosystem that connects the high Andean forest and páramo, where fragmentation makes the species more vulnerable to climate change. To answer the question of whether five related blueberry species could physiologically behave similarly in the dry season or if, on the contrary, they differ in their adaptability, three sites in Boyacá‐Colombia were climatically characterized to identify variables associated with soil water tension (Ψ_m_). Stem water potential (SWP) and leaf gas exchange traits were measured in the wet and dry seasons to evaluate water stress responses. Cumulative precipitation was the only climatic variable significantly correlated with Ψ_m_, and the linear regression of SWP as a function of Ψ_m_ had a moderate explanatory power (23.6%), suggesting the modulation of stem water potential despite soil irrigation. All species showed increases in leaf temperature and reductions in SWP, photosynthetic rate (A), and stomatal conductance (G_S_) during the dry season. However, species differed in their responses to drought with respect to water vapor deficit (VPD_L_), water‐use efficiency (WUE), the CO_2_ concentration ratio (Ci/Ca), and the severity of foliar symptoms. Although local specific climatic or edaphic conditions must be considered, we proposed a ranking from highest to lowest drought‐stress tolerance as follows: *Macleania hirtiflora* > *Vaccinium meridionale* > *Cavendishia pubescens* > *Macleania rupestris* > *Cavendishia bracteata.* Our results showed how specialization and adaptation to limiting conditions in the Andes are more strongly influenced by ecological pressures than by phylogenetic relatedness.

## 1 Introduction

The Ericaceae family is globally distributed in 4426 species and 129 genera (Luteyn 2002; Schwery et al. 2015; Stevens et al. 2004). A significant diversification occurred in mountain environments, particularly in the transitional zone between high‐Andean forests and páramo, known as the Ericaceae belt (Schwery et al. 2015). This ecosystem stands out for its biodiversity and structural heterogeneity, including a mixture of shrub and tree species arranged in multistratified patterns (Llambí 2015; Ramírez et al. 2022). It is subject to contrasting environmental conditions of low temperatures, high radiation, and strong winds, which vary significantly throughout the day, leading to episodes of water stress (Luteyn 2002; Rada et al. 2019; Ramírez et al. 2022). Additionally, with a narrow thermal niche range, high‐Andean biodiversity is among the most vulnerable to climate change (Cuesta et al. 2020; Tovar et al. 2022).

Water availability is a limiting factor determining species distribution (Basu et al. 2016). Drought stress imposes significant limitations on the physiological functioning of plants in both natural and cultivated ecosystems (Berg and Sheffield 2018; Grillakis 2019), with the soil water potential (Ψ_m_) serving as a valuable parameter for detecting stress conditions, since it measures the force plants must exert to extract water from the soil (Datta et al. 2017). Drought affects plant growth by reducing water potential, cell division, stomatal conductance, and photosynthetic capacity (Lambers and Oliveira 2019). Morphophysiological traits such as water‐use efficiency, leaf dry weight content, or stomatal traits are related to the capacity to tolerate water deficit (Becerra et al. 2022; Martínez‐Garza et al. 2013). Optimal Ψ_m_ thresholds for plants have primarily been evaluated for cultivated species (Klein 2004; Sadras and Milroy 1996; Xiao et al. 2023) and are largely unknown for most Andean plants.

The accumulation of local adaptations to the microclimate can lead to rapid speciation and adaptive evolution (Schwery et al. 2015). Colombia has the highest radiation of Neotropical Ericaceae, with nearly 60% of species being endemic (Pedraza‐Peñalosa et al. 2015), mainly found in Andean forests between 1000 and 3000 m in altitude (Luteyn 2002). The genera Macleania, Vaccinium, and Cavendishia are shrubs and trees that often coexist in these high‐altitude environments; they are characterized by edible berry‐like fruits, commonly known as Andean blueberries, and contribute to ecosystem services as water regulation, seed dispersion, and pollination, in addition to providing a productive alternative for local inhabitants (García‐Castro et al. 2023; Lagos‐Burbano et al. 2010; Luteyn and Pedraza 2008). The management, conservation, and restoration of plants in high‐Andean forests, especially in fragmented landscapes, requires a deeper understanding of plant ecophysiological vulnerability to water deficit and provides useful diagnostics in the face of climate change (Cruz and Lasso 2021).

Given that Ericaceae form a specific altitudinal ecosystem, it is important to question whether five closely related species from the tribe Vaccinieae have similar physiological performance in the dry season, or if they can be classified by their degree of vulnerability to water stress, detectable in the regulation of stem water potential (SWP) or leaf gas exchange. Therefore, this study set out three objectives: (1) to establish how climatic seasonality modulates soil water tension and how this, in turn, affects the SWP of five Andean shrub blueberry species, (2) to describe differences in physiological responses to the dry and wet seasons, and (3) to identify the most critical linear relationships that describe the particular vulnerabilities of these species to water stress.

## 2 Materials and Methods

### 2.1 Study Area

This study was conducted between September 2022 and April 2024 in fragmented areas of high‐Andean forest in the Colombian Eastern Cordillera, at elevations between 2700 and 3100 m (Boyacá department). The natural vegetation of the area is dominated by tree species such as *Quercus humboldtii* and *Alnus acuminata* and the genera *Miconia*, *Macleania*, *Cavendishia*, *Weimannia*, *Monochaetum*, and *Pentacalia*, among others. The daily temperature ranges from 4°C to 27°C. The forest fragments are influenced by nonexpansive anthropogenic disturbances such as livestock pastures, onion crops, and pine plantations. Therefore, they are key remnants for ecological recovery. Sites were chosen in three municipalities: Arcabuco (5°45′17.38″ N, 73°25′18.56″ W; multiannual mean rainfall of 1807 mm) and Ráquira (5°30′21.65″ N, 73°40′47.85″ W; multiannual mean rainfall of 985 mm) have a bimodal rainfall regime, with peaks from March to April and from October to November, and dry periods in January and from June to August. The third site was in Aquitania (5°25′40.66″ N, 72°54′14.57″ W; multiannual mean rainfall of 823.6 mm), which has a monomodal climatic regime with a rainy period from April to August and a dry season from December to February.

### 2.2 Study Species

Five species from the Ericaceae family, tribe Vaccinieae, were selected: *Vaccinium meridionale* and *Cavendishia bracteata* in Ráquira, *Cavendishia pubescens* and *Macleania hirtiflora* in Arcabuco, and *Macleania rupestris* in Aquitania. These blueberries grow in natural populations, which local communities harvest manually once or twice a year without using agricultural chemicals or technological treatments. Although three or four of these Ericaceae species coexist at the study sites, only populations with sufficiently close individuals were used for comparative physiological and microenvironmental measurements on the same day.

### 2.3 Climate and Microclimate

To depict seasonal variability and detect a possible recent intensification of drought, monthly rainfall data were obtained from the IDEAM National Catalogue of Stations (IDEAM 2024): 35160010‐Aquitania, 24010630‐Arcabuco, and 24010180‐Ráquira, spanning 1975 to 2022. The background period was defined as 1975 to 2007, and the recent period as 2008 to 2022. Additionally, during this study, one meteorological station (Davis Vantage Vue 6250) was installed at each site, recording rainfall, wind speed, temperature, and relative humidity from March 2023 to April 2024. The soil water tension, also known as matric potential (Ψ_m_), was measured using Irrometer SR tensiometers (one per site) installed at a depth of 30 cm, covering the layer that supports the higher root net of berry shrubs and is more susceptible to water depletion during drought. Measurements of Ψ_m_ were taken at three‐day intervals for 4 months during the dry season and 4 months during the wet season.

Microclimate data were collected 1 day per month at three time intervals: 08:00–09:30, 11:30–13:00, 15:00–16:30 in synchrony with the physiological measurements of blueberry plants. Digital thermohygrometers (Thermometer TA218 C/D) were used to measure relative humidity (%) and air temperature (°C) at 1 m foliage level. The set of microclimate variables was completed with records of photosynthetically active radiation (PAR; μmol m^−2^ s^−1^) from an IRGA (Li‐6800F, LI‐COR Biosciences, Lincoln, NE, USA).

### 2.4 Physiological Monitoring of Blueberry Plants

Five healthy plants per species were chosen for all physiological samplings. SWP (SWP) and gas exchange measurements were taken over 6 months, 1 day per month, spanning both dry and wet seasons, at three time intervals synchronized with microclimate readings. Given that, especially during the wet season, sporadic rainfall can make it impossible to use the equipment, three measurements at different times of day increase the likelihood of comparison with readings in the same schedule range in the dry season.

An apical branch from the middle stratum of foliage was selected from each plant, ensuring the leaves were adult and fully exposed. The branches were covered with hermetic aluminized bags 30 min before the water potential measurement to prevent transpiration and allow water balance between the leaves and the rest of the stem (Acevedo‐Opazo et al. 2010; Van Leeuwen et al. 2009). The branch or leaf was then cut from the bud and immediately placed in a Scholander pressure chamber (PMS Model 600), where the negative hydrostatic pressure, or tension in the xylem, was measured, corresponding to the SWP (MPa) (Gomes et al. 2014).

Because of their accessibility, five leaves from the lower and middle strata of foliage were selected, resulting in 15 measurements per shrub each sampling day. Leaf gas exchange variables included carbon assimilation (A, μmol m^−2^ s^−1^), stomatal conductance (G_S_, mmol m^−2^ s^−1^), transpiration rate (E, mmol m^−2^ s^−1^), CO2 intercellular/ambient CO_2_ ratio (Ci/Ca), vapor pressure deficit (VPD, kPa), water use efficiency (WUA, μmol CO_2_ mmol^−1^ H_2_O), PAR (μmol m^−2^ s^−1^), air temperature (Tair, °C), and leaf temperature (Tleaf, °C). Data were obtained using an IRGA portable photosynthesis system (Li‐6800F, LI‐COR Biosciences, Lincoln, NE, USA), setting a reference CO_2_ value of 400 μmol m^−2^ s^−1^ and a light point of 1800 μmol m^−2^ s^−1^ (Cruz and Lasso 2021).

### 2.5 Leaf Traits

The branches used for water potential measurements were assessed for health status, with particular attention to the severity of foliar diseases: chlorosis, necrosis, burning, and herbivory. The affected leaf area (LA) percentage was visually estimated for each symptom, and the symptom severity per individual plant was averaged based on the three branches. These leaves were stored in a humid chamber at low temperatures until further analysis was performed in the laboratory, using an AM300 LA meter (ADC‐BioScientific Ltd.). Three specific leaf area (SLA) measurements per individual plant were calculated as the fresh LA divided by the dry weight, obtained after 48 h in an oven at 70°C.

The morphometric characterization of stomata was performed on three healthy leaves per individual plant, which were stored in a humid chamber and refrigerated until laboratory analysis. Epidermal cuts were made, and three microphotographs were taken with a Leica ICC50W microscope camera at ×400 magnification, resulting in 45 microphotographs per species. ImageJ (v. 1.53 k) was used to precisely measure stomatal pore diameter and calculate stomatal density (SD) by counting the number of stomata in an area of 0.0713 mm^2^.

### 2.6 Statistical Analysis

The software Statistica v. 13 (TIBCO Software Inc.) was used to run all statistical analyses. The background monthly rainfall data from 1975 to 2007, as well as the number of dry months (cumulative rainfall lower than 5% of the monthly average) per year, were compared with records from the last 15 years (2008–2022) to assess if there has been an intensification of the dry season associated with climate change (two‐way ANOVA, with the site and period as factors). The variance of the data was calculated to visualize differences between sites and between the dry and wet seasons. The multivariate effects of the season and site on environmental conditions were analyzed using a Wilks test.

Spearman's correlation coefficients were calculated using monthly means to establish the linear relationships between climatic variables and soil water tension and how these relate to SWP. Linear or nonlinear models (GLZ) were used to maximize fit and explain dependencies between these variables.

The physiological traits of the five species were compared between dry and wet seasons using a two‐way analysis of variance (GLM univariate), with station and species as fixed factors. Pairwise mean differences were detected using the LSD post hoc test.

To examine how the relationships between SWP and other physiological gas exchange traits change between dry and wet seasons, Pearson's correlation coefficients were calculated, and differences between linear models were tested using one‐way analysis of covariance (ANOVA).

Finally, to understand the responses of the Andean blueberries to the dry‐wet seasonality, factors other than water tension, such as radiation, air temperature, and relative humidity, were considered. A correlation‐based principal component analysis (PCA) was applied to identify relationships between microenvironmental and physiological variable vectors (using the diurnal averages of physiological records per plant), highlighting the behavior of the five species along the resulting discriminant axes.

## 3 Results

### 3.1 Multiannual Rainfall and Climate Change

**FIGURE 1:**
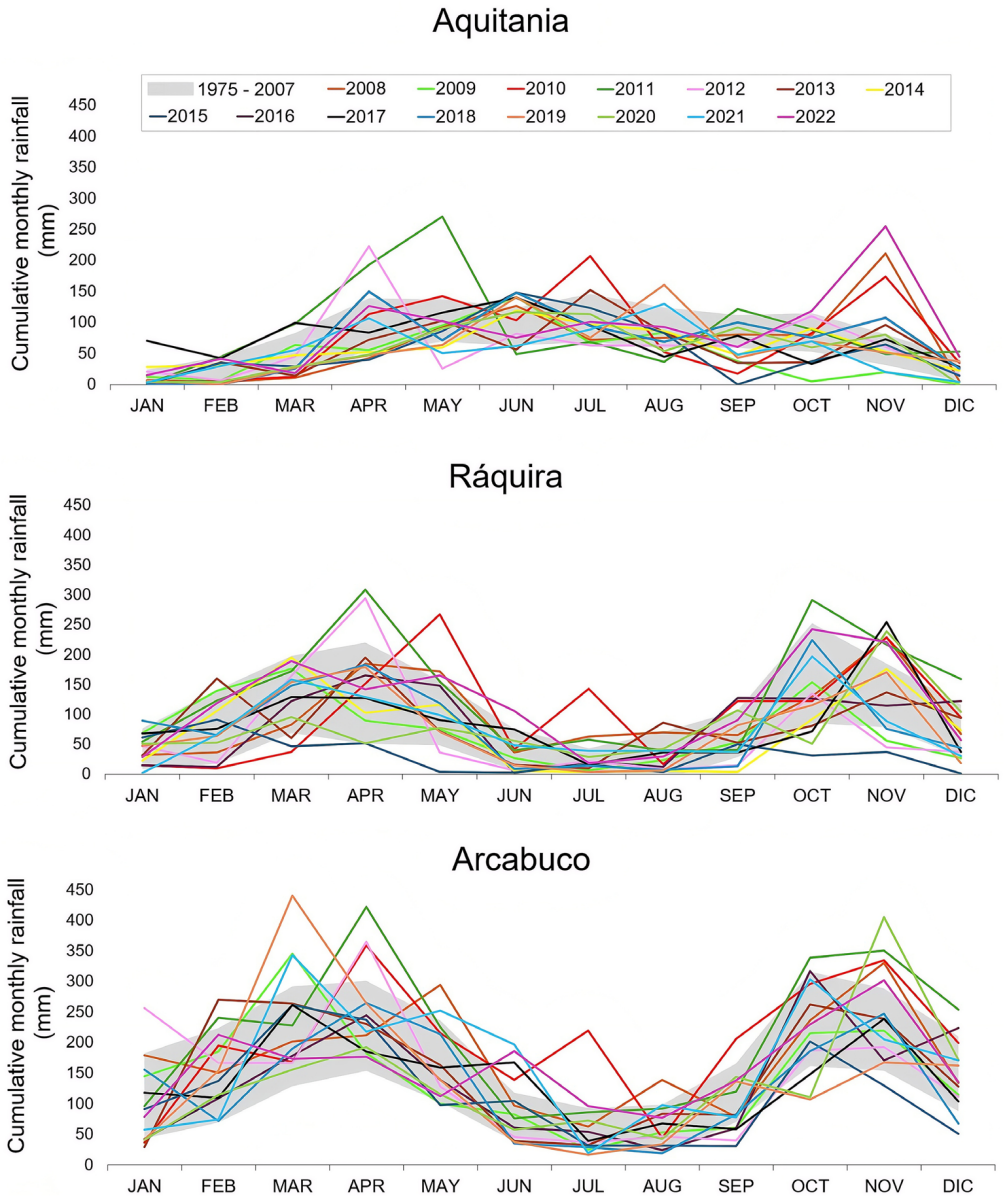
Comparison between the cumulative monthly rainfall of the background (min–max 2000–2007) and the recent period (2008–2022) in three sites of fragmented high Andean forest (Boyacá, Colombia).

The annual rainfall was significantly higher (*p* < 0.001) in Arcabuco (1800 ± 278 mm) compared to Ráquira (1031 ± 284 mm) and Aquitania (811.7 ± 132 mm), with no evidence (*p* = 0.672) of a change between the recent period (2008–2022) and the reference period (1975–2007). However, we found variations attributable to climate change, such as a recent and significant increase in the number of dry months per year (*p =* 0.019), from 4.2 to 5.2 (equivalent to 30 days) in Ráquira and from 3.5 to 4.1 (equivalent to 18 days) in Aquitania. The most notable changes in climatographs over the last 15 years are increased rainfall peaks, longer drought seasons, and a shift from unimodal to bimodal climate in Aquitania (Figure 1).

### 3.2 Environmental Variations Between Wet and Dry Seasons (2023–2024)

Marked variations in climatic conditions were observed across the study areas (Wilks multivariate test, *p* < 0.001; Table 1). Arcabuco recorded daily mean temperatures 2°C–7°C lower than Ráquira and Aquitania (Site effect: *p* < 0.001). Maximum and minimum temperatures occurred in Ráquira (27°C) and Aquitania (4.3°C). Aquitania showed the lowest relative humidity percentages with minimal variations throughout the year, while Ráquira and Arcabuco exhibited increases of about 30% during the wet season (Site effect: *p* < 0.001). The radiation reached its highest values in Aquitania (843.2 μmol m^−2^ s^−1^) and Ráquira (732.4 μmol m^−2^ s^−1^) during the dry season, while Arcabuco recorded low radiation during both climatic seasons (site effect: *p* < 0.001).

**TABLE 1:** Environmental variation (mean ± SE) between wet and dry seasons and among the three sites of study: Arcabuco, Ráquira, and Aquitania.

Season	Ráquira		Arcabuco		Aquitania	
	Dry	Wet	Dry	Wet	Dry	Wet
Monthly mean rainfall (mm) 2000–2022	31.75 ± 2.73	139.31 ± 6.71	72.83 ± 5.65	236.68 ± 8.08	25.75 ± 2.74	100.78 ± 5.32
Maximum air temperature (°C)	27.19 ± 0.5	27.41 ± 0.5	21.02 ± 0.5	20.74 ± 0.4	21.78 ± 0.6	18.82 ± 0.6
Minimum air temperature (°C)	6.5 ± 0.3	5.77 ± 0.4	7.8 ± 0.25	7.67 ± 0.3	4.33 ± 0.4	5.04 ± 0.5
Wind speed (m/s)	0.088 ± 0.01	0.058 ± 0.00	0.684 ± 0.04	0.403 ± 0.03	1.55 ± 0.03	1.49 ± 0.07
Diurnal air relative humidity (%)	45.01 ± 1.76	70.83 ± 2.18	53.48 ± 1.26	70.3 ± 1.83	39.11 ± 0.77	46.18 ± 0.48
Diurnal air temperature (°C)	23.32 ± 0.42	19.18 ± 0.48	16.94 ± 0.23	16.42 ± 0.23	21.06 ± 0.37	18.43 ± 0.24
PAR radiation (μmol m^−2^ s^−1^)	732.4 ± 110	591.4 ± 101	332.5 ± 27	452.5 ± 77	843.2 ± 78	651.4 ± 99
Soil water tension (kPa)	−37.76 ± 19.84	−7.39 ± 4.17	−25.04 ± 17.26	−4.0 ± 2.46	−14.24 ± 13.1	−3.14 ± 5.73

### 3.3 Soil Water Tension and SWP

Due to seasonal fluctuations in climatic and microclimatic variables, soil water tension varies substantially in the top 30 cm of soil depending on the season and study site (Site, Season, and Interaction Effect: *p* < 0.001). The highest soil water tension (Ψ_m_) was recorded in Ráquira, where the most significant change occurred, from −7.4 kPa in the wet season to −37.8 kPa in the dry season (Table 1). In contrast, Aquitania recorded the least negative Ψ_m_ values during both climatic seasons.

**FIGURE 2:**
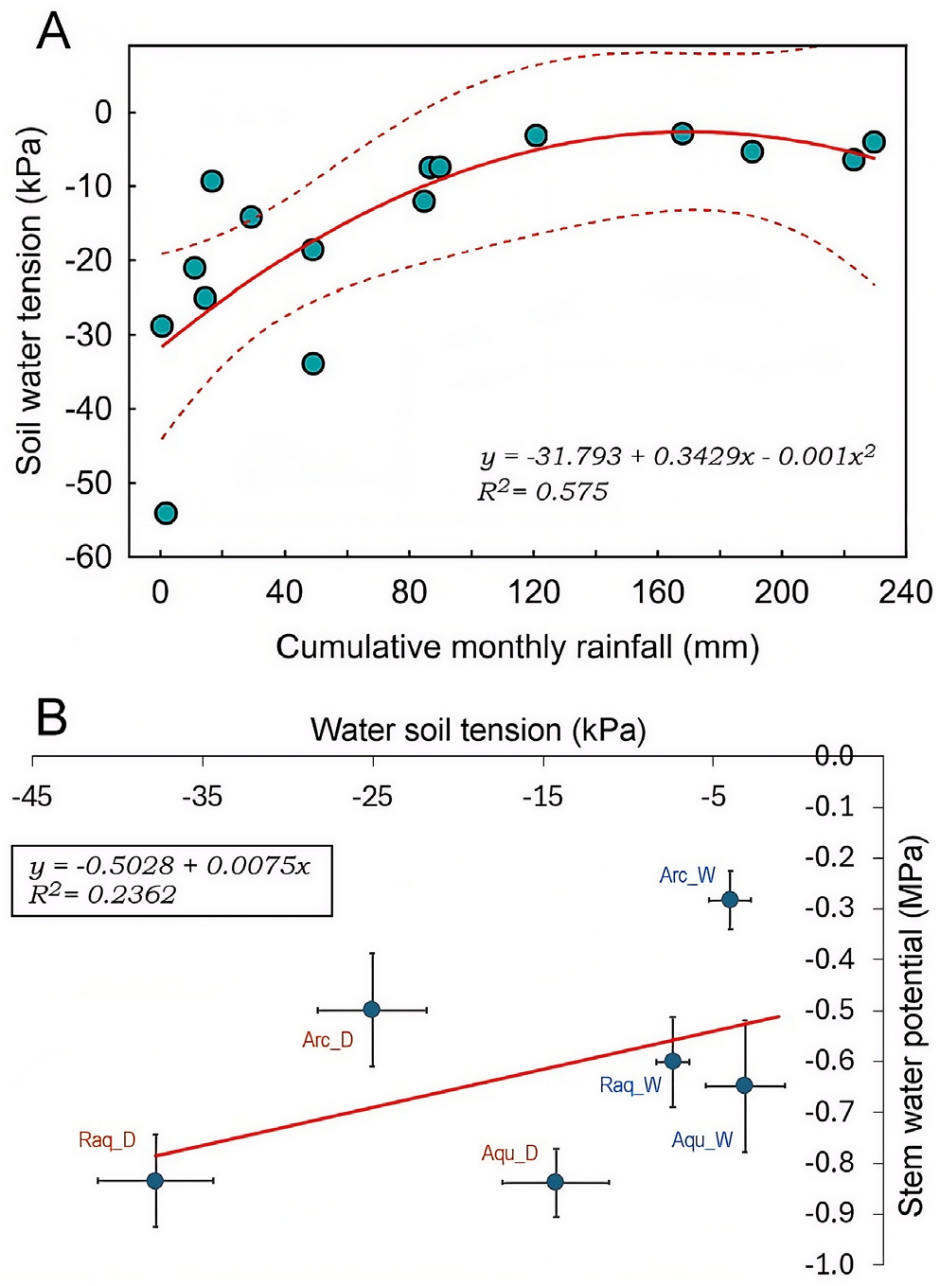
(A) Non‐linear model of average stem water potential as a function of cumulative monthly rainfall. (B) Linear relationship between stem water potential (SWP) and soil water tension (Ψm) in Arcabuco (Arc), Aquitania (Aqu), and Ráquira (Raq) during the wet (W) and dry (D) seasons. The spreads are 95% confidence intervals.

**FIGURE 3:**
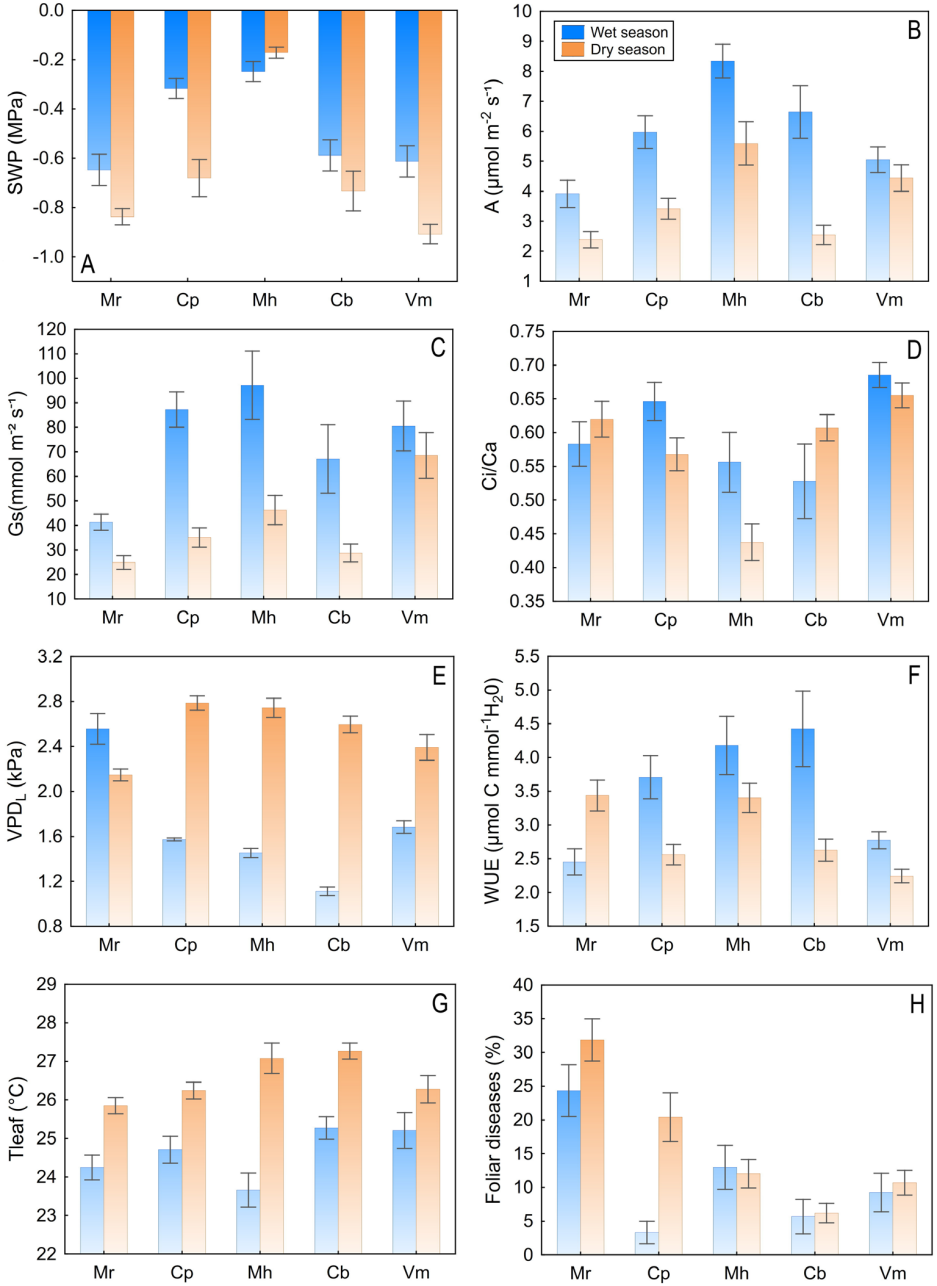
Physiological responses: (A) (stem water potential SWP, (B) carbon assimilation A, (C) stomatal conductance G_S_, (D) ratio of intracellular CO_2_ to ambient CO_2_ Ci/Ca, (E) leaf vapor deficit VPDL, (F) water‐use efficiency WUE, (G) leaf temperature T_leaf_, and (H) leaf disease severity) of five Andean berries to drought. The values represent the mean ± standard error calculated from five plants per species: *Macleania rupestris* (Mr), *Cavendishia pubescens* (Cp), *Macleania hirtiflora* (Mh), *Cavendishia bracteata* (Cb), and *Vaccinium meridionale* (Vm). Letters indicate differences between seasons and species (*p* < 0.05).

The only climatic variable that showed a linear relationship with soil water tension was the accumulated monthly precipitation (*r* = 0.673, *p* < 0.01). However, the most explanatory model (57.7%) for Ψ_m_ as a function of rainfall was polynomial (Figure 2A). The SWP also showed a linear relationship with Ψ_m_ (Figure 2B), although with a relatively low determination power (23.6%), indicating significant physiological adjustment capacity of plants to soil water deficiency. The most extreme values of SWP were −2.0 MPa in *C. pubescens* during the dry season and −0.02 MPa in *M. hirtiflora* during the wet season, both species from Arcabuco. However, *V. meridionale*, *M. rupestris* , and *C. bracteata* recorded the most negative means of SWP (−0.9, −0.8, and −0.75 MPa, respectively) during the dry season (Table 2 and Figure 3A).

**TABLE 2:** Physiological traits (mean ± SE) of five shrub and tree species of berries in the Andean forest from Boyacá, Colombia.

	Aquitania	Arcabuco		Ráquira	
	*M. rupestris*	*C. pubescens*	*M. hirtiflora*	*C. bracteata*	*V. meridionale*
A (μmol m^−2^ s^−1^)	2.96 ± 0.26	3.93 ± 0.33	6.61 ± 0.56	3.62 ± 0.53	4.68 ± 0.32
WUE (μmol CO_2_ mmol^−1^H_2_O)	3.03 ± 0.17	2.90 ± 0.16	3.76 ± 0.24	3.53 ± 0.33	2.51 ± 0.09
Ci/Ca	0.61 ± 0.02	0.58 ± 0.02	0.540 ± 0.01	0.48 ± 0.03	0.67 ± 0.003
G_S_ (mmol m^−2^ s^−1^)	31.07 ± 2.34	45.50 ± 4.65	65.10 ± 7.85	38.85 ± 5.87	73.20 ± 6.87
T_leaf_ (°C)	25.25 ± 0.20	27.94 ± 0.21	25.81 ± 0.43	26.74 ± 0.26	25.86 ± 0.30
VPD_L_ (kPa)	2.30 ± 0.06	2.54 ± 0.09	2.27 ± 0.13	2.20 ± 0.16	2.11 ± 0.10
SWP (MPa)	−0.723 ± 0.03	−0.528 ± 0.05	−0.280 ± 0.02	−0.730 ± 0.03	−0.701 ± 0.03
LA (cm^2^)	13.73 ± 1.31	44.65 ± 2.64	7.55 ± 0.66	3.41 ± 0.17	0.87 ± 0.1
SLA (cm^2^ g^−1^)	5.657 ± 0.77	5.493 ± 0.21	5.360 ± 0.46	5.313 ± 0.31	6.907 ± 0.17
Foliar diseases (%)	27.249 ± 2.08	16.98 ± 3.07	13.14 ± 1.85	5.46 ± 0.99	10.23 ± 1.06
SD (stomata mm^−2^)	139.94 ± 4.81	180.15 ± 6.37	181.70 ± 6.57	188.25 ± 5.21	290.17 ± 7.59

### 3.4 Physiological Traits of Leaves

The average carbon assimilation rate (A) was higher in the species *M. hirtiflora*, *C. pubescens* , and *C. bracteata* (8.2, 6.5, and 6.0 μmol m^−2^ s^−1^, respectively) during the wet season and significantly decreased (23%, 42%, and 65%) during the dry season for all three species (Figure 3B). The average stomatal conductance (G_S_) was higher in *M. hirtiflora*, *C. pubescens* , and *V. meridionale* during the wet season, reaching values between 80 and 95 mmol m^−2^ s^−1^, while it decreased by 50%, 59%, and 12.5%, respectively, during the dry season (Figure 3C). *M. hirtiflora* recorded the lowest Ci/Ca ratio during the dry season (0.44), and *C. bracteata* , in contrast to other species, showed a slightly higher Ci/Ca ratio in the dry season (0.6). During the dry season, an increase in the vapor pressure deficit (VPDL) and a decrease in water‐use efficiency were observed, except in *M. rupestris* (Figure 3F). The average leaf temperature ranged between 27.3°C in *M. hirtiflora* and 29.3°C in *M. rupestris* (Table 2). However, when discriminating by climatic seasons, the highest leaf temperatures were recorded in *M. hirtiflora* and *C. bracteata* during the dry season (Figure 3G). Foliar disease severity differed more among species than among seasons. Only *C. pubescens* showed higher leaf damage during the dry season (Figure 3H).

Regarding species‐specific foliar traits that could explain species' adaptive capacities to water stress, LA followed the order *C. pubescens* > *M. rupestris* > *M. hirtiflora* > *C. bracteata* > *V. meridionale*. Despite these differences, SLA was similar for all species. *V. meridionale* exhibited the highest SD; the SD in species of *Cavendishia* was similar, and the *Macleania* species significantly differed in the average SD. The largest and smallest stomatal pore diameters were found in *C. pubescens* and *V. meridionale*, respectively.

**FIGURE 4:**
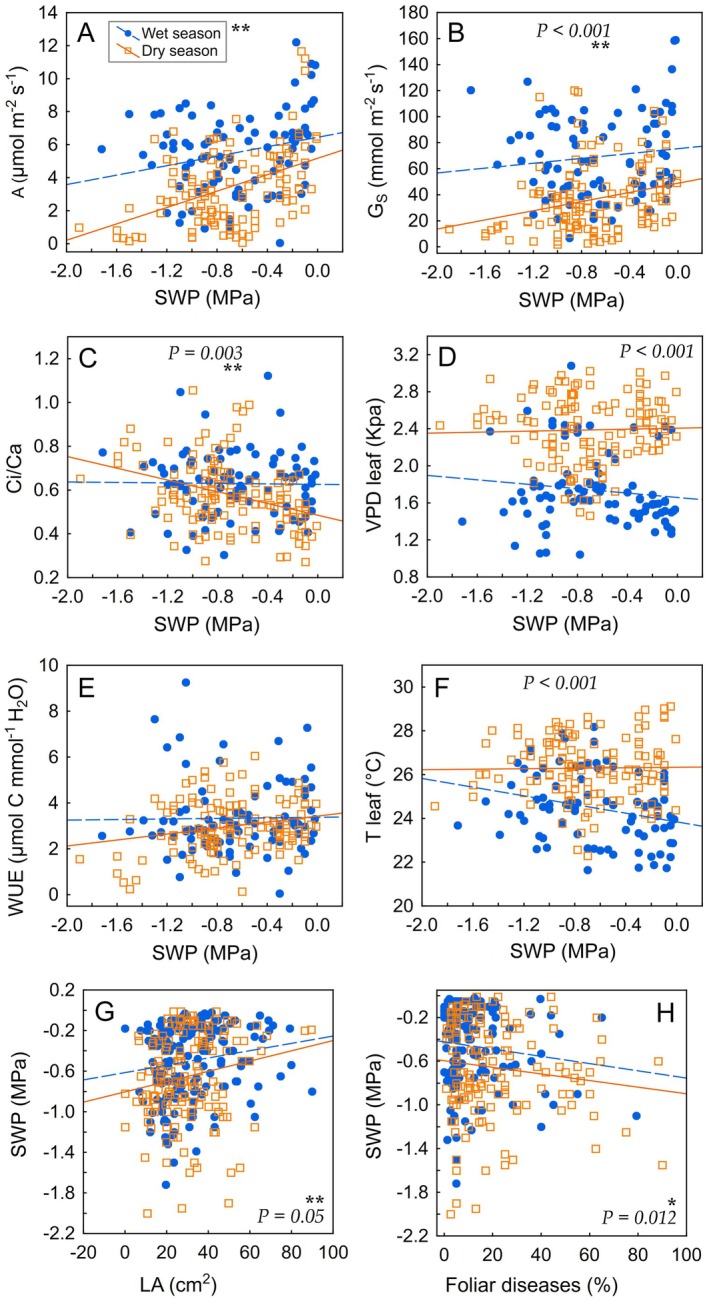
General linear relationships between stem water potential (SWP) and physiological (A–F) and leaf traits (G and H) variables of blueberries from the high Andean forest during the dry season (orange, *N* = 84) and the wet season (blue, *N* = 60). The leaf area and foliar diseases are shown as potential determinant variables on SWP (long‐term reaction). *p*‐Values depict significant effects of climate. Asterisks indicate a significant effect of the *X*‐axis covariate at *p* < 0.05 (*) or *p* < 0.01 (**).

All physiological variables had some linear relationship (generalized or by species) with SWP (Table 3 and Figure 4). Transpiration, carbon assimilation, and stomatal conductance showed the strongest significant correlations with SWP, indicating that limiting conditions (more negative SWP values) are associated with reduced photosynthesis and stomatal closure. *C. pubescens* was the species physiologically most susceptible to variations in SWP. Seasonality led to differences in the linear patterns of these relationships across all physiological variables, except for A and WUE, suggesting that plants capable of modulating SWP could reduce the photosynthetic limitations expected during drought. On the other hand, the analysis of covariances detected only a significant interaction (*p* = 0.04) between the climatic season and soil water stress on the Ci/Ca carbon concentration ratio, which may reflect specific variations in internal carbon concentration (Figure 3D).

**TABLE 3:** Pearson's product–moment correlation coefficients between physiological variables of leaves and soil water tension in five berries from the high Andean forest.

	Aquitania	Arcabuco		Ráquira		Overall
	*M. rupestris*, *N* = 36	*C. pubescens* , *N* = 36	*M. hirtiflora*, *N* = 36	*C. bracteata* , *N* = 36	*V. meridionale*, *N* = 36	*N* = 180
A (μmol m^−2^ s^−1^)	0.001	0.618*	0.300	−0.175	−0.088	0.366*
E (mmol m^−2^ s^−1^)	0.010	0.594*	0.313	0.532*	−0.043	0.272*
WUE (μmol CO_2_ mmol^−1^ H_2_O)	0.039	0.430*	−0.180	−0.500	−0.065	0.143
Ci/Ca	−0.042	−0.149	0.257	0.425	−0.006	−0.211*
G_S_ (mmol m^−2^ s^−1^)	0.039	0.579*	0.349	0.009	−0.096	0.264*
VPD_L_ (kPa)	−0.112	−0.572*	−0.087	0.285	0.095	−0.128
T_leaf_ (°C)	−0.307*	−0.171	−0.284	−0.011	0.050	−0.124
LA (cm^2^)	0.220	0.109	−0.101	−0.331	−0.052	0.250*
Foliar diseases (%)	−0.124	−0.213	0.411*	0.005	0.271	−0.133

* *p* < 0.05.

**FIGURE 5:**
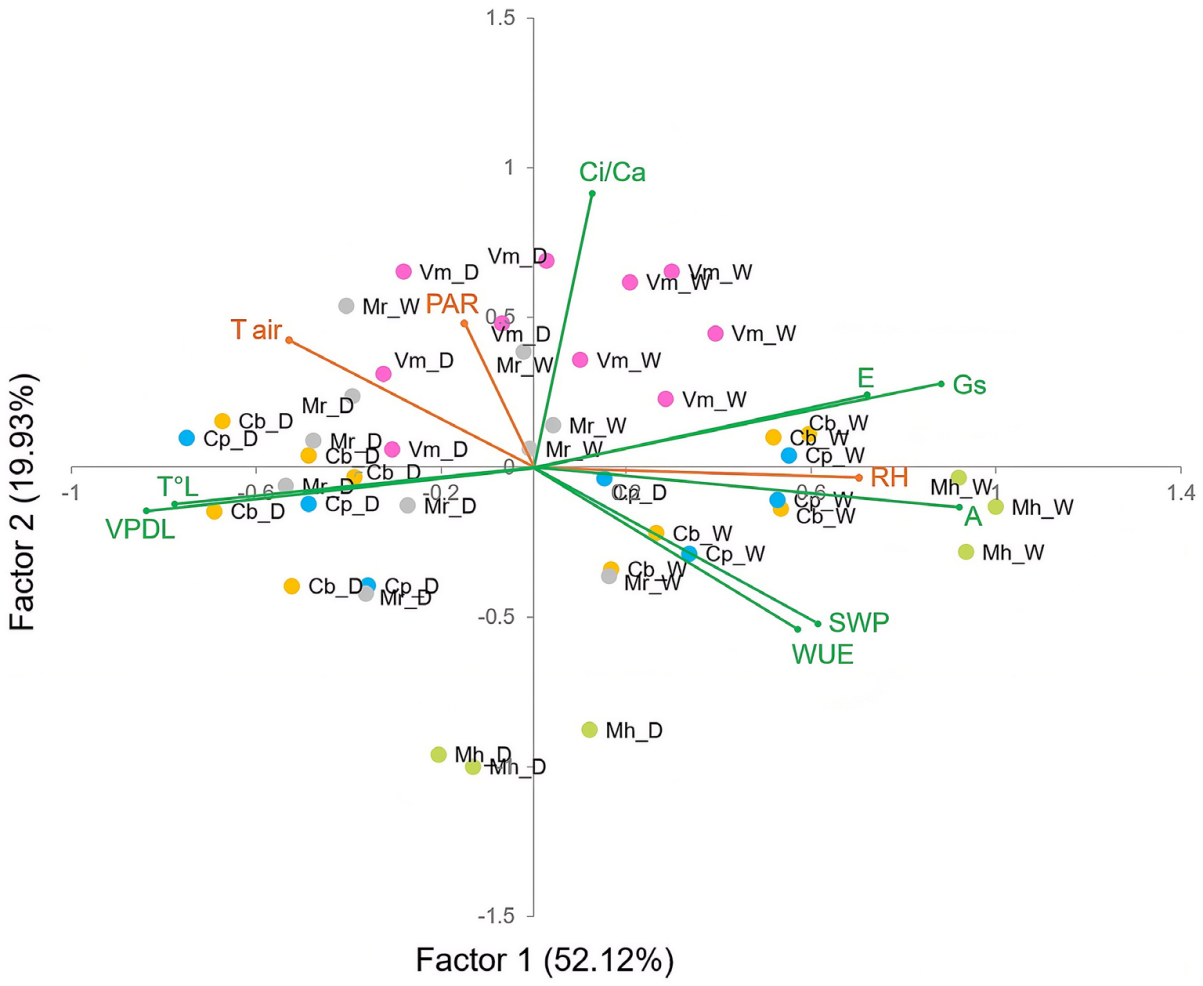
Principal component analysis (PCA) of Andean berry shrubs based on the variance in environmental variables (orange) and physiological traits (green) under wet (W) and dry (D) conditions. The first two components explained a joint variance of 72.1%. Arrows indicate the strength of the trait influence on the first two PCs. The extension of vectors indicates the contribution of each component to the PCA. Cb: *Cavendishia bracteata*, Cp: *Cavendishia pubescens*, Mh: *Macleania hirtiflora*, Mr: *Macleania rupestris*, Vm: *Vaccinium meridionale*. A, carbon assimilation; Ci/Ca, ratio of intracellular CO_2_ to ambient; E: transpiration; G_S_, stomatal conductance; PAR: photosynthetically active radiation, RH, air relative humidity; T_air_, air temperature; T_leaf_, leaf temperature; SWP, stem water potential; VPDL, leaf vapor deficit; WUE, water use efficiency.

Regarding other diurnal environmental factors, in addition to soil water tension, that may influence the multivariate patterns of physiological responses, the first two principal components of the PCA explained 72% of the total variance. The first component (52.12%) was strongly related to air relative humidity and partially to air temperature, while the second component (19.93%) showed partial relationships with PAR and air temperature. The physiological variables A, G_S_, and WUE were associated with high relative humidity values, while the Ci/Ca ratio was more associated with radiation. The first component discriminated the physiological behavior of plants in wet and dry seasons, with the genus *Cavendishia* being especially vulnerable, as their plants separate at opposite ends of this axis. *M. hirtiflora* is associated with low radiation environments, and its physiological response during the dry season is not influenced by drastic changes in air temperature or relative humidity, making it the species with the best water use efficiency. *V. meridionale* shows environmental affinities opposite to those of *M. hirtiflora*, tolerating more open environments, and the minimal separation between points suggests a better capacity for adaptation to climatic variations than the species of *Cavendishia*. Lastly, *M. rupestris* shows behaviors associated with both components, being susceptible to high temperatures and high leaf water vapor deficits (Figure 5).

## 4 Discussion

This study focused on five Ericaceae species within the same subfamily (Vaccinioideae) and tribe (Vaccinieae), where the genera *Cavendishia* and *Macleania* are genetically concentrated in a cluster described by Kron et al. (2002) as the “Andean Clade.” The authors note that the absence of a 12‐base‐pair sequence (*matK m*) places *V. meridionale* in an unresolved external clade, highlighting its distribution limited to Andean ecosystems in Colombia, Peru, Venezuela, and Ecuador. Using phylogenetic relationships as a framework for physiological behavior to drought, similar patterns would be expected within the same genera, with relatively distinct responses for *V. meridionale*. In our study, specific differences in how each species copes with water stress during dry seasons appear independent of their evolutionary proximity, which supports the idea that rapid speciation driven by environmental heterogeneity is primarily responsible for the adaptive radiation observed in the Andes (Schwery et al. 2015; Homeier et al. 2021).

The substantial variability in environmental conditions of the Colombian high‐Andean ecosystems reflects the climatic contrast between dry and wet seasons (Benavides‐Duque and Hernández‐Rodríguez 2022), leading to recent changes in species distribution, abundance, phenology, and physiology (Franco‐Vidal et al. 2013). Multiannual rainfall monitoring (Figure 1) reveals an intensification of wet and dry periods in the study area over the past 15 years. This trend is most pronounced in Aquitania and aligns with findings of a 19% increase in annual rainfall and a rise in average temperatures of 1°C–1.5°C (Armenta‐Porras 2020).

During wet seasons, soil water tension ranged from −3 to −7.8 kPa, indicative of optimal blueberry irrigation (Kim et al. 2011). In contrast, during the dry season in Ráquira, extreme Ψ_m_ averages of −37 kPa were recorded, which could be attributed to low cumulative monthly rainfall (Figure 2A), high temperatures, and elevated evaporative demand (González‐Rodríguez et al. 2016). An optimal Ψ_m_ for the development of blueberries and other fruits ranges between −4 and −8 kPa, with an irrigation threshold in −15 kPa, and initial signs of physiological depression in −25 kPa (Haman et al. 1997; Kim et al. 2011; Klein 2004; Sadras and Milroy 1996). In Ráquira and Arcabuco, where the threshold of −20 kPa was overcome during the dry season, the four species in these sites exhibited, on average, a decline in stomatal conductance and an increase in VPD of around 46%, accompanied by a rise in foliar temperature of 2°C. Nevertheless, the assimilation rate of these species was depressed by 38%, showing some capacity for physiological adjustment to drought (Figure 3).

Although it was expected that a substantial reduction of SWP would result from high soil water tensions, linear models showed that Ψ_m_ only explained 23.6% of SWP variability (Figure 2B), enforcing the idea of physiological adjustments. Critical leaf water potential values for stomatal closure in commercial blueberries ( *Vaccinium corymbosum* ) have been reported as −0.6 MPa by Bryla and Strik (2007) and −1.94 MPa by Rho et al. (2012). This study provides the first measurement of water potential in branches of high‐Andean shrub blueberries, recording extreme values between −2.0 MPa in *C. pubescens* during the dry season and −0.02 MPa in *M. hirtiflora* during the wet season. The correlation between A and SWP (Figure 4A) illustrates how, at least partially, soil irrigation and leaf water status limit productivity (Llambí et al. 2003; Rada et al. 2019; Sarmiento 2000).

The evaporative demand of blueberries was similar to that of other Andean species (Motzer et al. 2005). Contrasting with the behavior of the sclerophyllous shrub *Hypericum laricifolium*, where stomatal closure occurs at a VPD of 0.7 kPa (Sandoval et al. 2019), values of G_S_ closer to zero were extremely infrequent in our study. Despite water availability significantly influencing G_S_ (Table 3, Figure 4B), the hypostomatic nature of the five species minimizes water loss by reducing stomatal exposure to light and wind (Drake et al. 2019). Considering the overall low SLA, our results support the idea that Ericaceae species in the mountains achieve a higher tolerance to drought throughout their coriaceous, lignified leaves that require less turgescence water and confer resistance to cold‐induced diseases (Lütz 2010; Schwery et al. 2015; Zhang et al. 2012). This would allow them to maintain photosynthesis even under extreme midday SWP (−1.5 MPa) and improve water‐use efficiency (Cruz and Lasso 2021; Ramírez et al. 2022; Schwery et al. 2015).

The genus *Macleania* exhibited the highest WUE during the dry season (Figure 3F), a key adaptation to dry soils attributed to an isohydric regulation strategy that stabilizes the leaf water potential (Azócar and Rada 2006; Franks et al. 2007). However, *M. rupestris* exhibited the lowest mean G_S_, apparently due to low SD (Barral 2019) and high incidence of foliar disease, which could reduce assimilation rates (Grimmer et al. 2012). In *C. pubescens* , a burning leaf disease intensified during the dry season, which could allow to physiological deterioration (Table 2, Figure 3), agreeing with previous findings that leaves affected by foliar diseases and insect infestation tend to have a more negative leaf water potential, with possible effects on photosynthesis (Figure 4A,H) (Anderegg et al. 2015; Desprez‐Loustau et al. 2006).

Throughout a high, nonaltered by drought G_S_, *V. meridionale* demonstrated photosynthetic adaptability to high radiation, temperature, and water tension, which may be linked to its high SD (Barral 2019; Cáceres and Rada 2011) (Table 2 and Figure 3B). However, similar to *M. rupestris* , its lowest WUE makes the species susceptible to low soil irrigation. Semideciduous behavior was observed in the field during the dry season and may be a controlled strategy to reduce the risk of cavitation and embolism while maintaining stomatal conductance (La Rosa et al. 2017; Rabaey et al. 2006). In contrast, the two *Cavendishia* species had the lowest assimilation rates during the dry season, mainly attributable to a 60% decline in stomatal conductance. These species were strongly affected by a multivariate component of high leaf temperatures and severe VPD (Figure 5).

The species with the highest assimilation rate was *Macleania hirtiflora*, even under drought conditions, where efficiency declined by 33%. It is impossible to fully explain how this species maintains carbon fixation despite a 52% reduction in G_S,_ but apparently, a modulation toward low Ci/Ca supports the carbon gradient, thereby avoiding photosynthetic inhibition. One foliar trait that differs from that of its relative, *M. rupestris* , is pubescence; future studies could explore possible adaptations at the level of boundary‐layer resistance, osmotic adjustment, or the conservation of relative water content in leaves (Bickford 2016). On the other hand, we must highlight that this species is restricted to the stable environment of Arcabuco, where extremes in radiation, temperature, and soil water tension differ from those in Ráquira.

Excluding the behavioral patterns of plants adapted to extreme environments, mesophytic species exhibiting tolerant physiological responses during dry periods generally regulate leaf water potentials, maintain photosynthesis (although reduced), avoiding total stomatal closure, and display enzymatic optimization, reflected in reduced internal carbon concentrations, thereby sustaining the CO_2_ diffusion gradient. Additionally, by the persistence of water flux, these plants prevent excessive leaf heating and modulate VPD. Collectively, these responses result in high water‐use efficiency and reduced severity of foliar symptoms commonly associated with photooxidative stress (Gimeno et al. 2009; Grossiord et al. 2020; Lambers and Oliveira 2019; Soares‐Jancoski et al. 2022). Based on this convergence of “expected” drought‐tolerance features, and despite possible site‐specific masks, the comparative summary of the five studied species indicates that *M. hirtiflora* fulfills the greatest number of these optimal physiological traits, outperforming the others. Using a qualitative scoring approach, in which a positive value is assigned to traits consistent with the expected response and a negative value to traits opposite to it, the species can be ranked from highest to lowest drought stress tolerance as follows: *M. hirtiflora* > *V. meridionale* > *C. pubescens* > *M. rupestris* > *C. bracteata* (Table 4).

**TABLE 4:** Comparative physiological responses of the five Andean blueberry species during the dry season.

Expected physiological behavior during the dry season		*M. rupestris*	*C. pubescens*	*M. hirtiflora*	*C. bracteata*	*V. meridionale*
SWP (MPa)	High	−1		+1		−1
A (μmol m^−2^ s^−1^)	High	−1		+1	−1	+1
G_S_ (mmol m^−2^ s^−1^)	High	−1		+1	−1	+1
Ci/Ca	Low	−1	+1	+1	−1	−1
WUE (μmol CO_2_ mmol^−1^ H_2_O)	High	+1		+1		−1
VPD_L_ (kPa)	Low	+1	−1	−1		+1
T_leaf_ (°C)	Low	+1		−1	−1	
Foliar diseases (%)	Low	−1	−1		+1	+1
Total score		−2	−1	3	−3	1

*Note:* Species closest to expected drought‐tolerant behavior received a positive rating, while those with responses opposite to expectations received a negative rating. When the averages for the different physiological parameters were intermediate, no sign was assigned. The ratings summarize the behaviors observed during the dry season, as shown in Figure 3, and the total scores are the sum of positive and negative scores for each species.

The two macleanias, which, in a recent taxonomic review, have been considered the same species (Luteyn 2024), exemplify how specialization and adaptation to limiting conditions in the Andes are influenced more by ecological pressures than by phylogenetic relatedness. Nevertheless, in our study, physiological performance was context‐dependent under site‐specific conditions, and intrinsic adaptability could not be fully addressed by the current design.

## Funding

This research was funded through Call No. 14‐2021 of the Ministerio de Ciencia, Tecnología e Innovación (MINCIENCIAS), allocation of the Sistema General de Regalías de Colombia (SGR), under the project “Nursery sources and adaptability studies for the implementation of wild fruits as a strategy for ecological restoration and sustainable use in high Andean forest ecosystems in the department of Boyacá (BPIN2021000100295‐SGI3377).

## Conflicts of Interest

The authors declare no conflicts of interest.

## Data Availability

The data that support the findings of this study are openly available in Blueberries Dry season BOY at https://data.mendeley.com/datasets/h32v6nns6k/1.
